# Same school, different conduct: rates of multiple paternity vary within a mixed‐species breeding school of semi‐pelagic cichlid fish (*Cyprichromis* spp.)

**DOI:** 10.1002/ece3.1856

**Published:** 2015-12-08

**Authors:** Caleb Anderson, Alexandra Werdenig, Stephan Koblmüller, Kristina M. Sefc

**Affiliations:** ^1^ Institute of ZoologyUniversity of GrazUniversitätsplatz 28010GrazAustria

**Keywords:** Breeding systems, Lake Tanganyika, microsatellites, polyandry, sneaking

## Abstract

Mating system variability is known to exist between and within species, often due to environmental influences. An open question is whether, vice versa, similar environmental conditions entail congruent mating behavior, for example in terms of multiple paternity, in species or populations sharing largely comparable breeding modes. This study employed microsatellite markers to investigate the incidence of multiple paternity in *Cyprichromis coloratus* and *Cyprichromis leptosoma*, two sympatric, closely related, mouthbrooding Lake Tanganyika cichlids with similar ecological and behavioral characteristics including the formation of open‐water schools. Mouthbrooding females of both species were collected from the same mixed‐species breeding school at the same time, minimizing environmental variation during courtship and mating. In *C. coloratus*, four of 12 broods had more than one sire, with a mean of 1.33 reconstructed sires per brood. *C. leptosoma* exhibited multiple paternity in 18 of 22 broods, with a mean of 2.59 or 2.86 reconstructed sires per brood according to the programs gerud and colony, respectively. In addition, two broods were found to contain offspring transplanted from another brood. There was no significant difference in brood size between species, but mean sire number did differ significantly. Hence, substantial similarity in reproductive behavior along with shared environmental conditions during courtship and spawning did not lead to equal rates of polyandry or sneaking in the two species.

## Introduction

Multiple paternity of broods, clutches, and litters is a taxonomically widespread phenomenon (e.g., Pearse and Avise 2001; Avise et al. 2011; Coleman and Jones 2011; Taylor et al. 2014). Females often do not stand to gain any material benefits from mating with multiple males during a single reproductive cycle, but genetic benefits may arise that outweigh the costs of polyandry. For example, females may “trade up” by remating upon encountering higher quality males after an initial mating event, exercise postcopulatory choice by preferentially increasing the fertilization success of certain sperm, or reap the benefits of a bet‐hedging strategy which aims to increase offspring fitness via increased genetic diversity within broods (Arnqvist and Nilsson 2000; Jennions and Petrie 2000). Even alternative male reproductive tactics such as “sneaking,” which have generally been viewed as harmful to female fitness as they undermine female mate choice, may provide benefits to females in some circumstances (Reichard et al. 2007). From the male perspective, sneaking provides reproductive opportunities to individuals that fail to attract mates through courtship or competition and increases reproductive output of successfully mated males beyond the fecundity of their own mates (Taborsky 2008). The costs and benefits of sneaking and female‐driven polyandry depend on numerous factors including habitat characteristics, predation risk, mating opportunities, and genetic diversity of the population. As a result, rates of sneaking and polyandry vary with geographic, ecological, and demographic characteristics both between and within species (e.g., Griffith et al. 2002; Gosselin et al. 2005; Neff and Clare 2008; Mobley and Jones 2009; Candolin and Vlieger 2013). An open question is whether, vice versa, species or populations with similar breeding systems can be expected to exhibit similar rates of multiple paternity when mating takes place under comparable environmental conditions.

Fishes have been an attractive subject for parentage studies due to their diversity of breeding systems, including open‐water spawning, substrate spawning, mouthbrooding, and both female and male internal gestation (Coleman and Jones 2011), combined with the frequent occurrence of diverse alternative reproductive tactics (Taborsky 2008). Breeding systems are often initially described through behavioral observation, but genetic studies may reveal patterns of paternity that are challenging or impossible to observe directly. Genetic parentage analyses are most easily conducted when individual broods can be collected, but this is difficult when fish lack spatially confined reproductive behavior. This issue may be overcome for some species by employing exhaustive population sampling. For example, Serbezov et al. (2010) sampled an entire stream population of brown trout, a species lacking parental care behavior, and found that polygamous mating was common and both sexes exhibited large reproductive skew. However, open‐water species with planktonic offspring cannot be sampled in this way and collection of individual broods also is impossible. Thus, parentage analyses in open‐water fishes are generally restricted to species with reproductive systems that are conducive to brood sampling (Coleman and Jones 2011). For instance, the large ranges of shark species inhibit attempts to observe their breeding patterns, but parentage analyses are possible because they reproduce via internal fertilization, which enables the collection of entire broods with a known maternal genotype; genetic studies have demonstrated a high incidence of multiple paternity in many sharks including viviparous brown smoothhound sharks (Byrne and Avise 2012) and oviparous small‐spotted catsharks (Griffiths et al. 2012). Mouthbrooding species, such as many cichlids, also are conducive to brood sampling regardless of location because the entire brood is contained in the parent's buccal cavity. For example, Takahashi et al. (2012) were able to conduct a parentage analysis in the open‐water schooling *Xenotilapia rotundiventralis* by capturing mouthbrooding parents.

Cichlid fishes exhibit extended parental care of their offspring and many species form breeding pairs as well (Keenleyside 1991; Sefc 2011). Social behavior has been demonstrated to have an inconsistent relationship with genetic parentage in cichlids. For example, social monogamy concurred with genetic monogamy in several mouthbrooders and substrate spawners (Taylor et al. 2003; Egger et al. 2006; Schädelin et al. 2015), whereas other socially monogamous substrate spawners with biparental nest defense showed a high level of multiple paternity (Sunobe and Munehara 2003; Sefc et al. 2008). Rates of multiple paternity vary widely among polygynous mouthbrooding species (Kellogg et al. 1998; Haesler et al. 2011; Sefc et al. 2012), and intraspecific geographic and temporal variation in reproductive behavior was inferred from field observations and genetic parentage (Rossiter 1995; Matsumoto and Kohda 1998; Sefc et al. 2009). The two mouthbrooding *Cyprichromis* species addressed in this study occur in multispecies schools of up to several thousand individuals, synchronize spawning by the lunar cycle (Watanabe 2000; Takahashi and Hori 2006) and therefore experience similar environmental conditions during courtship and spawning. In the absence of environmental variation, paternity analyses are expected to reveal congruent mating systems in the two ecologically and behaviorally similar species.

*Cyprichromis coloratus* and *Cyprichromis leptosoma* are uniparental maternal mouthbrooders that breed in schools in the water column of Lake Tanganyika. Accounts by Konings (1998) and Takahashi and Hori (2006) describe segregation into three types of schools based on breeding status: mature males with ripe females, nonbrooding females with nonterritorial males, and mouthbrooding females by themselves. In contrast, the school from which samples were taken for the genetic parentage analysis in this study comprised both brooding and nonbrooding females as well as mature males of *C. leptosoma*,* C. coloratus* and *Paracyprichromis brieni*, which were segregated by species, sex and breeding status within the school. Both *C. coloratus* and *C. leptosoma* exhibit male polymorphism with respect to fin coloration, sexual dimorphism (colorful males, drab females) and an unusual mating strategy: males establish three‐dimensional territories in the water column several meters above rocky substrate into which they attempt to attract conspecific females (Konings 1998; Takahashi and Hori 2006). When spawning with a selected male, females first approach the male's genital papilla to collect sperm into their buccal cavities, and then release one or a few eggs at a time, which they immediately retrieve into their mouth. Neighboring territory holders trying to divert the females as well as sneaker males are common and threaten to parasitize on the selected males’ mating success (Konings 1998). If the observation that breeding female *Cyprichromis* often return to spawn with the primary chosen male despite being courted by multiple alternative males (Konings 1998) correlates with patterns of genetic parentage, we expect to see genetic contributions skewed toward a single male per brood. Furthermore, given that variation in mating behavior often correlates with environmental variation, the sharing of a common environment during courtship and spawning predicts congruent patterns of multiple paternity in the two species.

## Materials and Methods

### Field collection

All fish were caught from a school of *Cyprichromis* spp. in Lake Tanganyika, in front of “Kalambo Lodge” in Zambia (8°37′S, 31°37′E), during April 2012. The school occurred at a depth range of approximately 10–14 m. Fish were captured with the assistance of SCUBA. Gill nets were placed near the school, and fish were chased into the nets. Because mouthbrooding females often eject fry from their mouths when under severe stress, any females with offspring were placed into individual plastic bags to be taken to the surface. The remaining netted fish were kept in the nets and transported to the surface to be used for population samples. Caudal fin clips were collected from all adult fish including mouthbrooding females and preserved in 99% ethanol for later analysis. The captured broods (12 *C. coloratus* broods and 23 *C. leptosoma* broods) were euthanized and preserved in 99% ethanol as well. All adult fish were released after fin clips were collected.

### Laboratory analysis

DNA extraction was conducted using proteinase K digestion followed by protein precipitation with ammonium acetate for adult female fish and a standard Chelex 100 resin protocol (Walsh et al. 1991) for fry. All individuals were genotyped at 6 previously developed microsatellite loci: TmoM11 (Zardoya et al. 1996), UNH1009 (Carleton et al. 2002), UNH 2016 (Albertson et al. 2003), UME003 (Parker and Kornfield 1996), Ppun9 (Taylor et al. 2002), and UNH130 (Lee and Kocher 1996). Fragment amplification was conducted via polymerase chain reaction (PCR) using a fluorescent‐labeled forward primer (HEX, FAM, or NED). Two multiplex reactions (TmoM11, UNH1009, and UNH2016; UME003 and Ppun9) were used to amplify five of the six loci, while UNH 130 was amplified separately. The PCR parameters were as follows: 94°C for 5 min, 35 cycles of 94°C for 30 sec, 47°C (TmoM11, UNH1009, and UNH2016), or 50°C (UME003, Ppun9, and UNH130) for 1 min, and 72°C for 50 sec, and a final extension at 72°C for 7 min. Each PCR contained 1 *μ*L of adult fish DNA or 10 *μ*L of fry DNA and 0.5 *μ*mol/L of each primer for multiplexes or 1 *μ*mol/L for individual amplifications as well as the following: 10× reaction buffer (BioTherm, GeneCraft, Köln, Germany) with 15 mmol/L MgCl_2_ in the reaction, 1 U DNA polymerase (BioTherm), 62.5 *μ*mol/L of each dNTP, and water as needed to achieve a reaction volume of 20 *μ*L. PCRs were run on an Applied Biosystems 2720 thermal cycler, and the products were checked via gel electrophoresis using 2% agarose solution. All fragments were sized using an internal size standard (GeneScan‐500 ROX; Applied Biosystems) with an ABI 3130xl automatic sequencer (Applied Biosystems) and genemapper 3.7 software (Applied Biosystems, Vienna, Austria).

### Genetic data analysis

Microsatellite loci were characterized using 36 *C. coloratus* (12 mouthbrooding females and 24 population sample individuals) and 36 *C. leptosoma* (22 mouthbrooding females and 14 population sample individuals). Gene diversity estimates and tests for Hardy–Weinberg equilibrium were calculated in arlequin 3.5.1.2 (Excoffier et al. 2005). Additionally, loci were tested for heterozygote deficits (indicative of null alleles) with genepop 4.3 (Raymond and Rousset 1995). Exclusion probabilities were calculated in gerud 2.0 (Jones 2005). Paternal genotypes were reconstructed using gerud 2.0 (Jones 2005) and colony 1.2 (Jones and Wang 2010), programs which use different reconstruction methods that may produce different numbers of sires for a given brood. gerud uses offspring and maternal genotypes to determine the minimum possible number of fathers for those offspring and then finds the most likely configuration of paternal genotypes through a combination of population allele frequencies and Mendelian segregation. In contrast, colony uses a maximum likelihood model to cluster offspring into full‐sib and half‐sib families and reconstruct parental genotypes. Offspring genotypes were scored manually in GeneMapper, and PCR was replicated when a reconstructed sire was supported by only one or two alleles or when there was a mismatch between a mother and her offspring. After scoring errors were corrected, final analyses in colony were conducted with an error rate set to zero. identity (http://www.uni-graz.at/~sefck/identity4.exe) was used to search for the presence of identical sire genotypes reconstructed from different broods across both species. The search was limited to reconstructed sires that had contributed at least six offspring to a brood because smaller offspring numbers are not sufficiently informative about the sire's genotype (Sefc et al. 2009). The presence of skewed paternal contributions in the colony results for each brood was assessed using skew calculator 2003 (https://www.eeb.ucla.edu/Faculty/Nonacs/PI.html) to calculate the binomial skew index *B* (Nonacs 2000) and its associated *P*‐value using 10,000 simulations. A *B* value of zero indicated a random distribution of offspring among sires, while a significant positive value indicated skew and a significant negative value indicated an excessively even offspring distribution. Skew could only be calculated for broods with more than one sire using this method. Differences in rates of multiple paternity (numbers of sires per brood) and brood size (number of young per brood) between species and correlations between sire numbers and brood size were tested in generalized linear models (GLM) with negative binomial error distributions using the R package glmmADMB (Skaug et al. 2013). The difference in the proportion of multiply sired broods between species was tested in a GLM with a binomial error distribution. For all models, we report the parameter estimate *β* and its standard error, the test statistic *z*, and the corresponding *P*‐value.

## Results

The six microsatellite markers were highly polymorphic with high expected heterozygosity (Table 1). The sole deviation from Hardy–Weinberg equilibrium occurred at locus Ppun9 in *C. coloratus* (Table 1), but because observed heterozygosity at this locus was slightly greater than expected heterozygosity (*H*_o_ = 0.943 and *H*_e_ = 0.936) and genepop indicated no significant heterozygote deficit (H‐W exact test for heterozygote deficiency, *P* = 0.56), it is unlikely that null alleles would interfere with the paternity analysis. Across all six loci, exclusion probabilities (one parent known) were 99.99% and 99.98% for *C. coloratus* and *C. leptosoma*, respectively (Table 1).

**Table 1:** Characterization of microsatellite loci used in paternity analysis of *C. coloratus* and *C. leptosoma*

Locus	H_E_		HWE		*E*_1_	
	*coloratus*	*leptosoma*	*coloratus*	*leptosoma*	*coloratus*	*leptosoma*
TmoM11	0.94	0.59	0.62	0.34	0.857	0.428
UNH1009	0.94	0.90	0.13	0.20	0.871	0.786
UNH2016	0.96	0.95	0.91	0.99	0.902	0.901
UME003	0.95	0.92	0.65	0.21	0.872	0.821
Ppun9	0.94	0.91	0.04	0.47	0.855	0.811
UNH130	0.86	0.67	0.39	0.08	0.702	0.463
All Loci	Mean 0.93	Mean 0.82	–	–	0.9999	0.9998

*H*_E_, expected heterozygosity; HWE, *P*‐value for Hardy–Weinberg equilibrium test; *E*1, exclusion probability when one parent is known.

**Figure 1:**
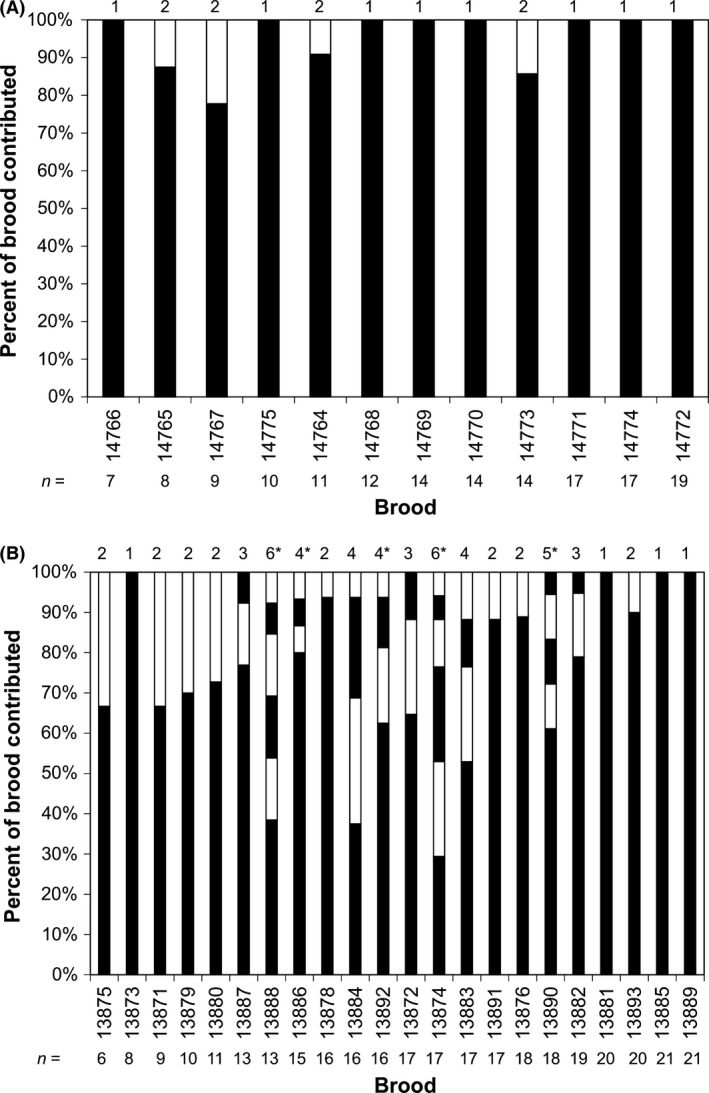
Brood contributions from the reconstructed sires in colony: A, *C. coloratus*; B, *C. leptosoma*. Numbers above each bar show number of sires per brood, with an asterisk indicating that the number of sires in the gerud analysis was one fewer than the colony result. *n*, number of offspring in the brood.

*Cyprichromis leptosoma* and *C. coloratus* differed significantly in the proportion of multiply sired broods (81.8% and 33.3%, respectively; GLM, *β *= 2.20 ± 0.83 for *C. leptosoma* compared to *C. coloratus*,* z* = 2.66, *P* = 0.008). The mean number of reconstructed sires per brood was 1.33 in *C. coloratus* (gerud and colony) and 2.59 (gerud) or 2.86 (colony) in *C. leptosoma*. Both methods reconstructed the same number of sires with the exception of five broods in *C. leptosoma*, and these exceptions always resulted in a difference of just one sire, so only the colony reconstructions are shown in Fig. 1. As GERUD underestimates and COLONY overestimates true sire numbers, when markers are not sufficiently informative, but converge on the true number, when applied to highly informative datasets, the congruency between the two methods lends strong support to the present sire number reconstructions (Sefc and Koblmüller 2009). Sire number per brood differed significantly between species for both gerud (GLM, *β *= 0.664 ± 0.283 for *C. leptosoma* compared to *C. coloratus*,* z* = 2.34, *P* = 0.019) and colony reconstructions (GLM, *β *= 0.764 ± 0.281 for *C. leptosoma* compared to *C. coloratus*,* z* = 2.72, *P* = 0.0064). There was no significant interspecific difference in brood size (mean and standard deviations: 15.4 ± 4.3 in *C. leptosoma*, 12.7 ± 3.8 in *C. coloratus*; GLM, *β *= 0.193 ± 0.104 for *C. leptosoma* compared to *C. coloratus*,* z* = 1.86, *P* = 0.067), and brood size was not correlated with sire number within species (colony output; GLM: *C. coloratus*,* β *= −0.041 ± 0.070, *z* = −0.59, *P* = 0.560; *C. leptosoma*,* β *= 0.009 ± 0.031, *z* = 0.30, *P* = 0.762) or across both species (GLM: *β *= 0.024 ± 0.027, *z* = 0.89, *P* = 0.370). None of the reconstructed sire genotypes occurred in more than one brood, suggesting that reproductive success is shared among multiple males in each species. Paternal contributions were skewed toward a major or exclusive sire in most broods (Table 2, Fig. 1). Notable exceptions occurred in *C. leptosoma*, where some broods were sired by multiple males none of which contributed more than 53% of offspring (Fig. 1).

**Table 2:** Paternity reconstruction results in *C. coloratus* and *C. leptosoma*

Female ID	Number of genotyped offspring	# Sires (gerud)	# Sires (colony)	S1	S2	S3	S4	S5	S6	*B* value	*P*
*C. coloratus*											
14764	11	2	2	10	1					**0.289**	**0.014**
14765	8	2	2	7	1					0.219	0.078
14766	7	1	1	7						–	–
14767	9	2	2	7	2					0.099	0.177
14768	12	1	1	12						–	–
14769	14	1	1	14						–	–
14770	14	1	1	14						–	–
14771	17	1	1	17						–	–
14772	19	1	1	19						–	–
14773	14	2	2	12	2					**0.219**	**0.009**
14774	17	1	1	17						–	–
14775	10	1	1	10						–	–
*C. leptosoma*											
13871	9	2	2	6	3					0.000	0.503
13872	17	3	3	11	4	2				**0.115**	**0.020**
13873	8	1	1	8						–	–
13874	17	5	6	5	4	4	2	1	1	0.002	0.443
13875	6	2	2	4	2					−0.028	0.686
13876	18	3	3	16	2					**0.062**	**0.004**
13878	16	2	2	15	1					**0.352**	**0.000**
13879	10	2	2	7	3					0.030	0.356
13880	11	2	2	8	3					0.058	0.203
13881	20	1	1	20						–	–
13882	19	3	3	15	3	1				**0.283**	**0.000**
13883	17	4	4	9	4	2	2			0.069	0.051
13884	16	4	4	6	5	4	1			0.008	0.393
13885	21	1	1	21						–	–
13886	15	3	4	12	1	1	1			**0.353**	**0.001**
13887	13	3	3	10	2	1				**0.237**	**0.005**
13888	13	5	6	5	2	2	2	1	1	0.000	0.508
13889	21	1	1	21						–	–
13890	18	4	5	11	2	2	2	1		**0.169**	**0.002**
13891	17	2	2	15	2					**0.263**	**0.001**
13892	16	3	4	10	3	2	1			**0.148**	**0.006**
13893	20	2	2	18	2					**0.295**	**0.001**

Number of offspring per sire (S1–S6) are derived from colony reconstructions. Binomial skew index (*B*) values with corresponding significant *P* values are bolded. Broods with only one sire cannot be tested for skewed distributions of paternity.

Four *C. leptosoma* offspring had alleles that did not match the maternal genotype. Two of these fry had a mismatch at one locus (locus UNH1009 in brood 13873; locus UNH2016 in brood 13874), a pattern likely indicative of mutation rather than incorrectly assigned maternity. The remaining two mismatched fry are more likely a result of brood mixing. One fry (brood 13886) had mismatches at four loci (TmoM11, UNH1009, UNH2016, UME003), but its genotype was matched with another mother in the dataset (13893) and it was found to be a full‐sib to 18 of the 20 offspring in that mother's brood (Fig. 1B, Sire 1 offspring). This relationship suggests that the fry was transplanted from the biological mother to the observed mother, and it was therefore excluded from the paternity analysis of the brood in which it was collected. The transplanted offspring was at approximately the same developmental stage as the other fry in the observed mother's brood, with a yolk sac still visible. Because these fry were not yet free‐swimming, the transplant may have taken place during spawning, but this cannot be confirmed in the absence of behavioral data. The fourth mismatched fry (brood 13872) was incongruent with the maternal genotype at three loci (UNH1009, UME003, Ppun9), its genotype did not match with any other mothers in the data set, and it was excluded from the paternity analysis. The sampled mothers are a small proportion of breeding mothers in the school, so it is likely that the true mother was present in the school, but not collected for sampling.

## Discussion

Perhaps the most surprising finding of this study is the significant difference in multiple paternity rates between two behaviorally and ecologically similar as well as closely related species such as *C. coloratus* and *C. leptosoma*. Mating system variability is often associated with geographic or temporal variation in environmental conditions (e.g., Twiss et al. 2007; Mobley and Jones 2009; Sefc et al. 2009; Candolin and Vlieger 2013). However, the broods analyzed in this study were collected at the same time from the same school, and as both *Cyprichromis* species appear to synchronize their spawning with the lunar cycle (Watanabe 2000; Takahashi and Hori 2006), the ecological differences generally thought to produce such variability were not present. Alternative sources of variation are related to species‐specific reproductive, demographic or behavioral traits. For instance, clutch size can contribute to multiple paternity rates, particularly in the context of bet‐hedging (Jennions and Petrie 2000), but there was no significant difference in brood size between the two *Cyprichromis* species. A more likely source of mating pattern variation are potential between‐species differences in the densities of males and females within the school, which can influence mating patterns (Reichard et al. 2004; Neff and Clare 2008) as well as sneaking and defense strategies. Unfortunately, bad visibility during sampling prohibited the collection of demographic data from the school.

There are multiple nonexclusive potential explanations for the occurrence of polyandry in *Cyprichromis*, one of which is the presence of male alternative reproductive tactics. Konings (1998) observed that both neighboring territory holders and intruding sneaker males tried to lure spawning *Cyprichromis* females into collecting their sperm, while the females’ mates violently expelled potential sneakers from their territories. In our analysis, most of the *C. coloratus* and *C. leptosoma* broods were skewed toward the contributions of a primary or exclusive sire. This pattern is consistent with Konings’ (1998) observation that females are not susceptible to diversions by nonpreferred males and furthermore suggests that attempts of sneaking are rewarded with only moderate fertilization success per brood.

Multiple paternity can also reflect females seeking benefits from mating with more than one male (Arnqvist and Nilsson 2000; Jennions and Petrie 2000; Taylor et al. 2014). Because *Cyprichromis* are planktivorous and females only remain in a male's territory long enough to spawn (Konings 1998), the provisioning of direct benefits such as food resources or defense from predators (Yasui 2001) is unlikely to explain female‐driven polyandry in these species. Alternatively, a female might mate multiply simply to ensure that she receives a sufficient amount of sperm to fertilize her entire clutch of eggs (Barlow 2000). However, in mouthbrooding species that have relatively small clutches such as *C. coloratus* and *C. leptosoma*, the enclosed space of the female buccal cavity and the small number of eggs to be fertilized suggest that fertilization insurance is not a primary cause of multiple paternity (Immler and Taborsky 2009). A trade‐up strategy, according to which females should first mate indiscriminately to ensure fertilization but then select high‐quality males for subsequent matings, can help females to maximize offspring quality when potential mates are encountered sequentially and simultaneous comparisons are therefore not possible (Pitcher et al. 2003). In *Cyprichromis*, however, females select mates from among a dense aggregation of courting males, which allows for direct comparisons and generates little incentive to accept a nonpreferred male for part of her brood. Multiple mating can also serve to increase the offspring's genetic diversity and as a bet‐hedging strategy against suboptimal mate choice. Immler and Taborsky (2009) suggested that multiple mating of females aiming to maximize the genetic diversity within their brood should result in a positive correlation between brood size and number of sires. There was no significant correlation found in either *C. coloratus* or *C. leptosoma*, so shopping for diversity is an unlikely explanation for the observed multiple paternity patterns. Bet‐hedging is predicted to arise under conditions such as small population size, an unstable environment, and low cost of remating (Yasui 2001). Only the latter condition may be fulfilled in the *Cyprichromis* schools, whereas the typically high number of individuals within schools (Konings 1998) and their localization in the stable shallow layers of Lake Tanganyika (Coulter 1991) contradict the prediction.

In summary, considering observations of spawning behavior (Konings 1998) and distributions of paternity within broods (present study), sneaking seems to be the most likely source of multiple paternity of *Cyprichromis* broods. Importantly, equivalent ecological requirements, life histories, and breeding systems did not preclude different rates of multiple paternity in the two species despite shared environmental conditions during courtship and spawning. While this finding does by no means contradict the important influence of the environment on mating behavior in general, subtle variation in species‐specific properties likewise appears capable of inducing substantial variation in mating behavior.

## Conflict of Interest

None declared.
